# Correlation between Pancreatic Duct Variation and Related Diseases: An Effective Method Observing the Dual-Energy CT with Low-keV Monoenergetic Images

**DOI:** 10.3390/diagnostics13030520

**Published:** 2023-01-31

**Authors:** Ruike Zhang, Zhengying Li, Xiaoli Hu, Hongwei Liang, Gaowu Yan, Dan Xie, Jiao Zhang, Yongmei Li

**Affiliations:** 1 Department of Radiology, The First Affiliated Hospital of Chongqing Medical University, Chongqing 400016, China; 2 Department of Ultrasound, University-Town Hospital of Chongqing Medical University, Chongqing, 400016, China; 3 Department of Radiology, Suining Central Hospital, Suining 629000, China

**Keywords:** pancreatic duct variation, pancreas-related diseases, dual-energy CT, MRCP

## Abstract

Purpose: Pancreatic duct variation can affect the secretory function of the pancreas. We aimed to explore the pancreatic duct variation, observed using low-keV monoenergetic images [MEI (+)] of dual-energy CT (DECT), and its relationship with related diseases. We further sought to compare pancreatic duct imaging using low-keV MEI (+) of DECT and magnetic resonance cholangiopancreatography (MRCP). Materials and Methods: The DECT and MRCP images of 854 patients were evaluated retrospectively. The 808 patients’ pancreatic duct types were classified according to the anatomy and the opening of the pancreatic ducts, and the correlation with related diseases was analyzed. The DECT and MRCP images of 852 patients were graded according to the sharpness of the pancreatic ducts for evaluation. Results: A higher prevalence of acute pancreatitis (AP), chronic pancreatitis (CP), and duodenal papillary carcinoma (DPC) was observed in the variant group. Of the 27 AP cases in the variant group, 9 patients (33.3%) were Type 3c. Additionally, Type 4a was significantly correlated with AP and CP (*p* < 0.05). Low-keV MEI (+) of DECT outperformed the MRCP images in the sharpness of the pancreatic ducts in 852 patients. Conclusions: Pancreatic duct variation is associated with AP, CP, and DPC. Low-keV MEI (+) DECT is an effective method to observe the pancreatic duct system.

## 1. Introduction

The pancreas is the second largest digestive gland in the human body and plays an important role in the process of digestion. With the morbidity of pancreatic disease increasing significantly [1,2,3], further in-depth and detailed research on the etiology of the pancreas and related diseases is needed. Most previous etiological studies have focused on external causes, such as long-term smoking and alcoholism, and some internal causes, including heredity, genetics, and race [1,2,3,4,5]. Recently, some researchers have suggested that the anatomical variation of the pancreatic duct may be connected with some pancreatic diseases [6], a potential factor that is easily ignored.

The pancreas consists of two parts: the exocrine pancreas and the endocrine islets. The exocrine pancreas consists of most of the gland and pancreatic ducts [7]. As an important secretory channel of the pancreas, the variation, obstruction, and injury of the pancreatic duct system can affect the balance of endocrine and exocrine function [8,9,10]. The exocrine pancreas is also affected by distinct diseases, such as acute and chronic pancreatitis; most pancreatic cancers are ductal carcinomas [11]. In the clinic, most congenital pancreatic duct variations are diagnosed accidentally after the occurrence of pancreas-related diseases. Some studies have shown that pancreas divisum (PD) tends to induce recurrent AP and CP [12]; in contrast, some researchers hold that the frequency of pancreatitis does not differ between patients with PD and unaffected individuals [13]. Thus, the congenital anatomical variation of the pancreatic duct and its relationship with pancreatic-related diseases remain unclear [14]. Meanwhile, few studies have focused on other types of pancreatic duct variation.

In the choice of observation methods, endoscopic retrograde cholangiopancreatography (ERCP) has clear defects; it is an invasive examination that often causes postoperative complications and may further aggravate the condition [15,16]. Subsequently, magnetic resonance cholangiopancreatography (MRCP) gradually replaced the former because of its noninvasive characteristics [6]. However, MRCP has some limitations: it reduces spatial resolution, making it less sensitive to certain abnormal anatomy or lesions; visualization is affected when peripancreatic exudation or peritoneal fluid is present; and the assessment of ampullary lesions is somewhat constrained [17,18,19]. Given the limitations of many examinations, DECT may be a promising method. Early research has shown that using multislice spiral CT could easily identify congenital variations of the pancreatic duct and pancreas [20]. In recent years, with the rapid development and popularization of dual-energy CT technology, low-keV MEI (+) of DECT has provided better contrast for the observation of low-density pancreatic lesions while using the lowest radiation dose and amount of contrast medium possible [21,22]. In the non-secretin enhanced scan, the unenhanced pancreatic duct showed low-density imaging, which can provide a clearer course of the pancreatic duct for diagnostics, thus improving the diagnostic efficiency.

The aim of this study is to explore the relationship between the types of pancreatic duct variation and pancreas-related diseases by low-keV MEI (+) of DECT. Moreover, we subjectively evaluated the sharpness of the pancreatic ducts between low-keV DECT and MRCP, hoping to provide better choices and supplements for clinicians in terms of examination methods.

## 2. Materials and Methods

### 2.1. Subjects

All patients were treated at the First Affiliated Hospital of Chongqing Medical University. The radiology information system/picture archiving and communication system (RIS/PACS) of our hospital was utilized to search and identify patients. We retrospectively evaluated images of 854 patients who underwent abdominal DECT and MRCP within 2 weeks before treatment between July 2020 and September 2022. These patients were confirmed by clinicopathology (biopsy or surgical section) and/or serological indicators (serum amylase, etc.). 2 patients with recurrence after surgical resection of pancreatic neoplasms were excluded (the pancreatic duct was already incomplete), and 852 patients’ DECT and MRCP images were graded according to the sharpness of the pancreatic ducts. Forty-four patients with poor quality of either DECT or MRCP images were excluded from the study. Finally, 808 patients’ pancreatic duct types were classified according to the anatomy and the opening of pancreatic ducts, and the correlation with related diseases was analyzed.

**Figure 1:**
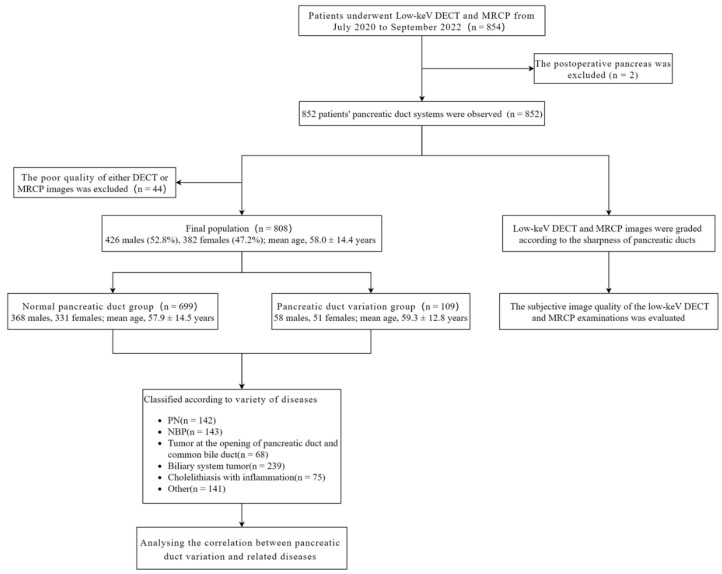
Flow chart of the study population. PN: Pancreatic neoplasms; NBP: Nonbiliary pancreatitis; DECT, Dual-energy computed tomography; MRCP, Magnetic resonance cholangiopancreatography.

Figure 1 shows the flow chart of the study population.

### 2.2. Imaging Analysis—Anatomical Classification of Pancreatic Ducts and Related Disease Grouping

All CT and MRCP images were consistently evaluated by two experienced abdominal radiologists (8–12 years of experience) on PACS workstations. The radiologists were blinded to the clinical information of any patient. Any discrepancies were resolved by a third more experienced radiologist.

The anatomical variation of the pancreatic duct was divided into 5 groups according to the classification of AnaDugic et al. To facilitate better description and typing, the main pancreatic duct (MPD) in this paper refers to the anatomically large and functionally dominant duct, which is not related to its embryonic origin [6]. This is slightly different from the definition of MPD in traditional anatomy.

**Figure 2:**
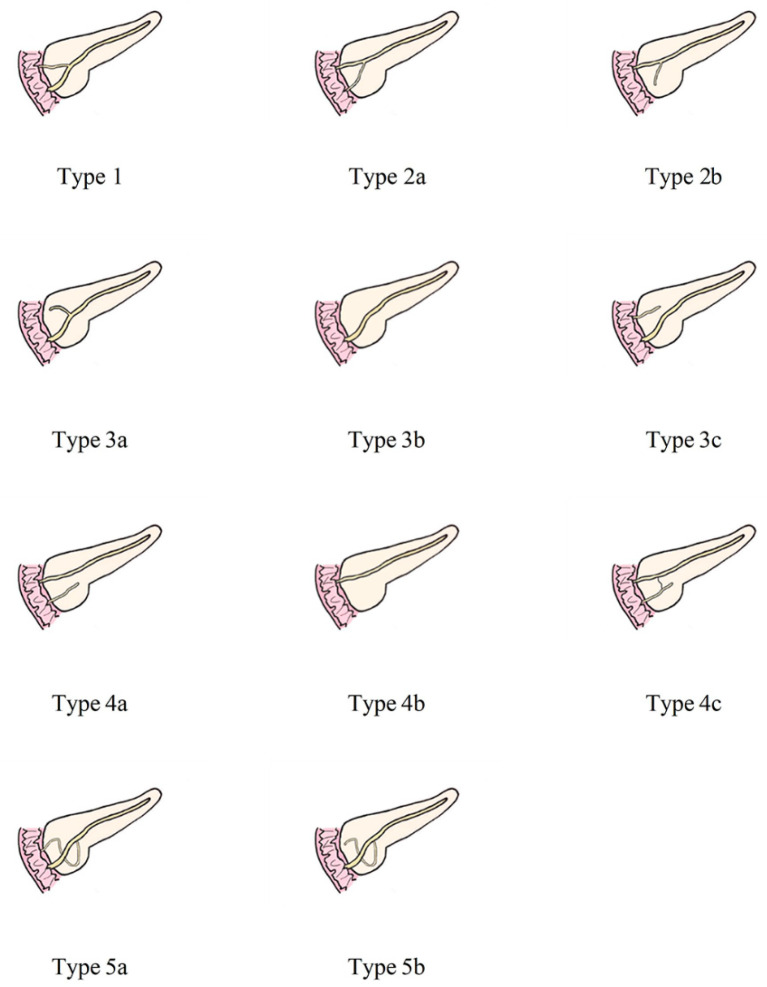
Anatomical classification of pancreatic duct system. Type 1: MPD drainage to MP, APD drainage to MiP, local communication; Type 2: MPD drainage to MiP, APD drainage or non-drainage to MP, local communication; Type 3: Subtype a, MPD drainage to MP, APD non-drainage to MiP, local communication; Subtype b, only MPD drainage to MP, but no APD; MPD drainage to MP and non-communicating APD drainage to MiP are type 3c, which is called “reverse pancreas divisum”; Type 4: Pancreas divisum. Subtype a- “complete pancreas divisum”, MPD drainage to MiP, APD drainage to MP; Subtype b, only MPD drainage to MiP, but no APD; Subtype c- “incomplete pancreas divisum”, there is local communication based on subtype a; Type 5: Ansa pancreatica--MPD drainage to drainage to MP, and annular or S-shaped APD is indirectly connected to MPD through ventral small branches. MPD, main pancreatic duct; APD, accessory pancreatic duct; MP, major papilla; MiP, minor duodenal papilla.

Figure 2 shows the detailed classification of the pancreatic duct system.

In this study, types 1 and 3b were set as the normal group, and the remaining types 2, 3a, 3c, 4, and 5 were set as the variant group. In addition, we established groups of pancreas-related diseases and non-pancreas-related diseases. The pancreas-related diseases included groups of pancreatic neoplasms (PN), nonbiliary pancreatitis (NBP), duodenal papillary carcinoma (DPC), and ampullary carcinoma (AC). DPC and AC were added because the former is an important outflow channel of pancreatic juice, while the latter is closely related to the pancreatic parenchyma and the pancreatic duct in the head of the pancreas and may even have partial communication [23]. Pancreatic duct variation may affect the excretory function of the secretory channel and lead to the occurrence of lesions. The non-pancreas-related diseases include groups of biliary system tumors (except AC), cholelithiasis with inflammation, and other (no lesions in the biliopancreatic system and duodenum).

## 3. Imaging Technology

### 3.1. Enhanced Dual-Energy CT

The CT image data were collected on a DECT scanner (SOMATOM Force, Siemens Healthineers) in dual-energy mode through two X-ray tubes with different kilovolts (A tube, 100 kV; B tube, Sn150 kV). The high-voltage tube used a tin filter. The settings of the two scanners were as follows: collimation 128 × 0.6 mm; rotation time 0.5 s; and reference tube current time product of pitch 0.6× A tube, 260 mAs. Automatic exposure control (CAREDose4D) was used for all scans. The images were taken from the caudal direction from the hepatic fornix to the pubic symphysis, and the pancreas was scanned completely. A nonionic contrast medium (Ultravist 370 Bayer Healthcare; or Iopamiro 370 Healthcare) was injected through the vein around the forearm at a dose of 1.2 mL/kg at a flow rate of 3.5–5 mL/s, and 40 mL of saline was then rinsed at the same injection rate. Arterial phase scanning was delayed 10 s after the abdominal aorta reached the trigger threshold (100 HU), and portal venous phase scanning was performed 30 s after the end of the arterial phase [24].

The study mainly observed 40–50 keV MEI (+) reconstructions acquired by DECT, which previous studies have claimed better show low-density lesions of the abdominal parenchymal organs [24,25]. More intuitive images of the pancreatic duct system can be obtained using the maximum intensity projection (MIP) and the minimum intensity projection (MinIP).

### 3.2. MRCP

In our hospital, 1.5T (Signa HDx, GE Healthcare) and 3.0T (Magnetom Skyra, Siemens Healthcare GmbH) magnetic resonance imaging (MRI) scanners were used for the MRCP examination. Before the examination, the patients fasted for 10 h and refrained from drinking for 6 h. No intravenous or oral contrast agent was given.

T2-weighted single-shot half-Fourier acquisition fast spin–echo and 3D respiratory-triggered spin–echo images were achieved, with a section thickness of 3 mm, a flip angle of 180°, and a field of view of 256 mm × 256 mm. The best images of the bile duct and pancreatic duct were obtained by a thin-layer MRCP rotation at different angles. The images were obtained during breath holding, and the MIP images were analyzed to understand the probable anatomical changes from multiple angles [26].

The patients underwent both DECT and MRCP. However, the images focused more on the pancreatic duct itself than the diagnosis of the disease. To assess the sharpness of the pancreatic ducts, we established a set of grade standards. An image quality that failed to display and therefore seriously affected the diagnosis was graded as Grade 1. Grade 2 was considered when the image was unclear and needed another examination to reach the diagnosis, and Grade 3 was considered when the image was clear and could be diagnosed independently.

### 3.3. Statistical Analysis

All data were analyzed with SPSS 25.0 software. The quantitative (numerical) data were expressed as the means ± standard deviation (SD), and the qualitative (categorical) variables were expressed as numbers and percentages. Comparisons of the quantitative data were performed using student’s *t*-test. The analysis of the categorical data was performed using Fischer’s exact test and chi-squared test to classify and test the distribution of pancreas-related diseases in the normal and variant groups. Spearman’s rank order correlation was performed to verify differences between low-keV MEI (+) of DECT and MRCP in observing the image quality of the pancreatic duct system. *p* < 0.05 was considered statistically significant.

## 4. Results

### 4.1. Patient Characteristics

From July 2020 to September 2022, a total of 808 patients (mean age: 58.0 ± 14.4 y) completed both abdominal DECT and MRCP imaging, including 426 males (52.8%) and 382 females (47.2%). The variant group included 109 patients (mean age: 59.3 ± 12.8 y), with 58 males (53.2%) and 51 females (46.8%). The normal group included 699 patients (mean age: 57.9 ± 14.5 y), with 368 males (52.6%) and 331 females (47.4%). There were no significant differences between the normal group and the variant group regarding age or sex (*p* > 0.05). The variant group comprised 64 patients (58.7%) with pancreas-related diseases and 45 patients (41.3%) with non-pancreas-related diseases. The distribution of pancreas-related and non-pancreas-related diseases was statistically different between the two groups (*p* = 0.001). Patient characteristics and clinical data are summarized in Table 1.

### 4.2. Frequency of Pancreatic Duct Variation

**Figure 3:**
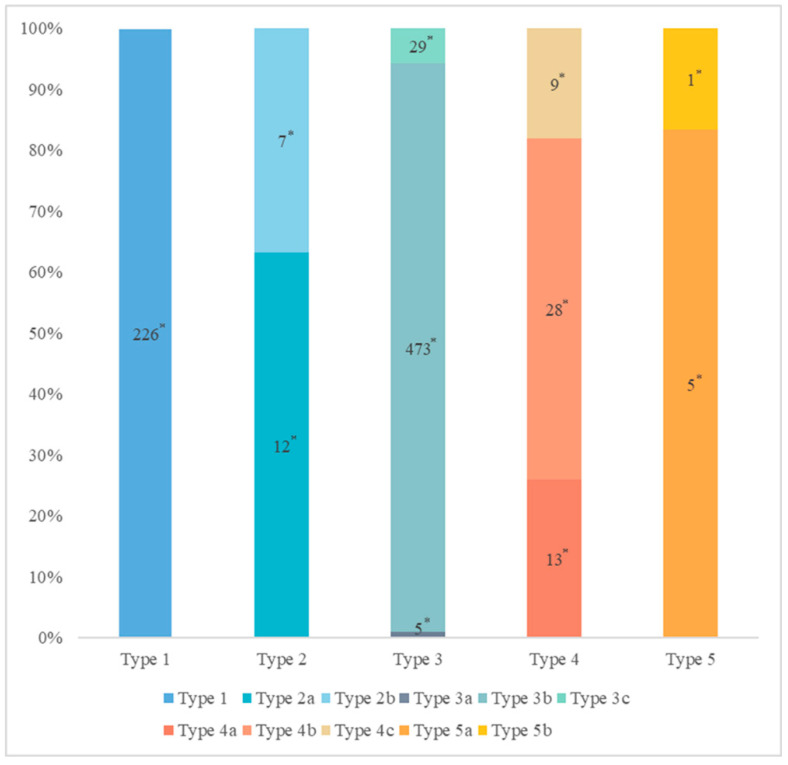
The distribution of pancreatic duct variation. * Quantity of subjects.

The distribution of pancreatic duct variation is shown in Figure 3.

**Figure 4:**
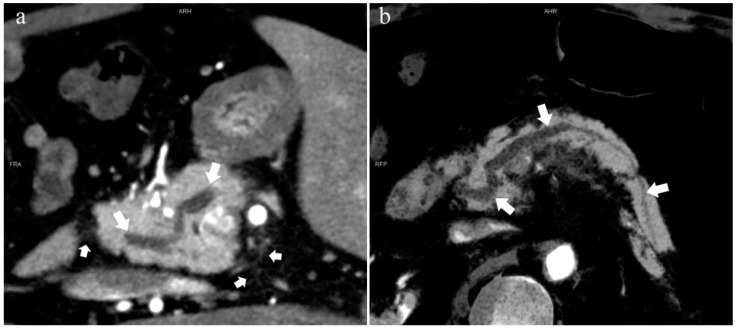
Type 3b, 63-year-old male patient with AP. (**a**): MIP [45 keV MEI (+)] shows an S-shaped dilated MPD (long arrow) in the head of the pancreas. APD is not displayed. The fat space around the head of the pancreas is blurred (short arrow). (**b**): MinIP [45 keV MEI (+)] clearly shows the overall shape of “S”-shaped MPD.

**Figure 5:**
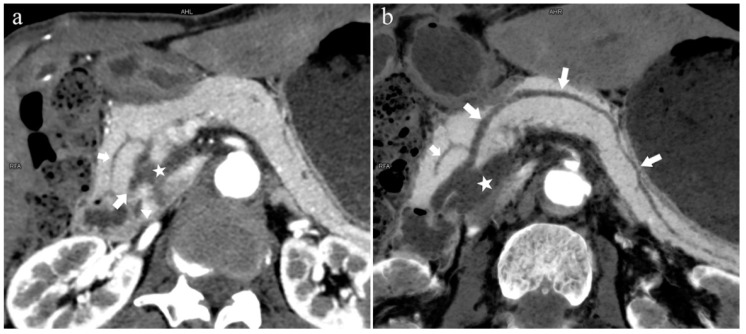
Type 1. A 68-year-old male patient with IPMN. (**a**): MIP [45 keV MEI (+)] shows a tumor (pentagram) and the dilated MPD (long arrow) in the head of the pancreas. APD (short arrow) drained from MiP and communicated with MPD. The common bile duct (arrow) is clearly visible. Postoperative pathology confirmed that the mass was IPMN. (**b**): MinIP [45 keV MEI (+)] clearly shows the overall direction of MPD.

The MPD flowed into the MP, and no APD was the most common anatomical type (type 3b, Figure 4) (58.5%, 473/808); the second type was a classical double division with the double duct structure (type 1, Figure 5) (28.0%, 226/808).

**Figure 6:**
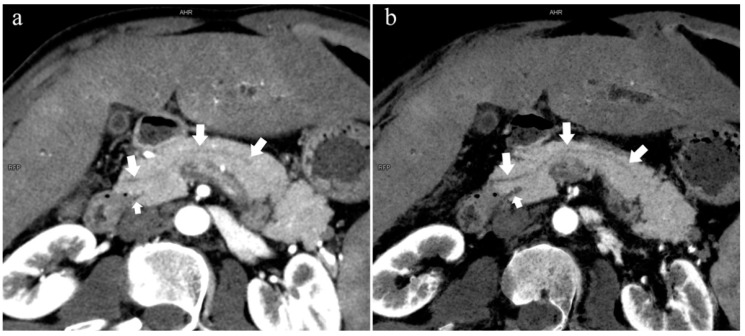
Type 4b. A 46-year-old male patient with hilar cholangiocarcinoma. (**a**): MIP [45 keV MEI (+)] shows that the MPD (long arrow) flows into the MiP and extends to the tail. APD is not shown. The initial part of the common bile duct (short arrow) is visible. (**b**): MinIP [45 keV MEI (+)] shows the course of the pancreatic duct more clearly. MPD refers to anatomically large and functionally dominant ducts.

**Figure 7:**
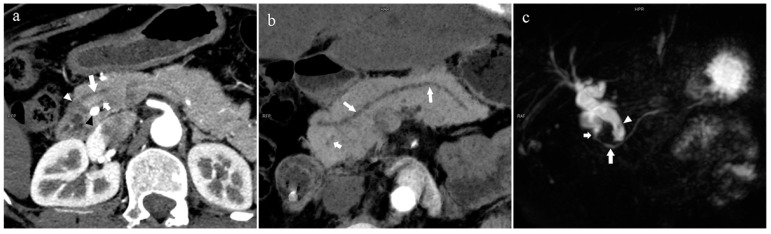
Complete pancreas division (Type 4a). A 42-year-old female patient with common bile duct stones. (**a**): MIP [45 keV MEI (+)] shows that the MPD (long arrow) flows into the MiP (arrowhead) and the APD (short arrow) flows into the MP. There is no communication between MPD and APD. The T-tube (black arrow) can be seen in MP and the common bile duct. (**b**): MinIP [45 keV MEI (+)] more intuitively shows the overall shape of MPD (long arrow) and APD (short arrow). MPD flows into MiP and extends to the tail of the pancreas. (**c**): Oblique coronal thick slab MRCP image shows MPD (long arrow) flows into the MiP (short arrow) and the APD is not displayed. The stone (arrowhead) is located in the middle and lower part of the common bile duct. MPD refers to anatomically large and functionally dominant ducts.

**Figure 8:**
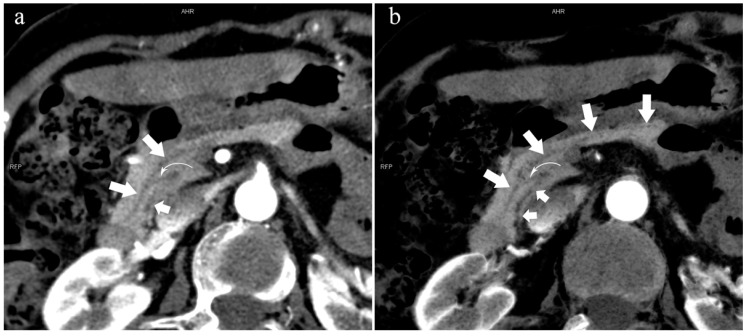
Type 4c. A 73-year-old female patient with multiple stones of the intrahepatic bile duct. (**a**): MIP [45 keV MEI (+)] shows that MPD (long arrow) flows into MiP, and APD (short arrow) flows into MP with a small communication branch (bent arrows) between them. (**b**): MinIP [45 keV MEI (+)] improves visualization of small pancreatic ducts and their branches. MPD refers to anatomically large and functionally dominant ducts.

**Figure 9:**
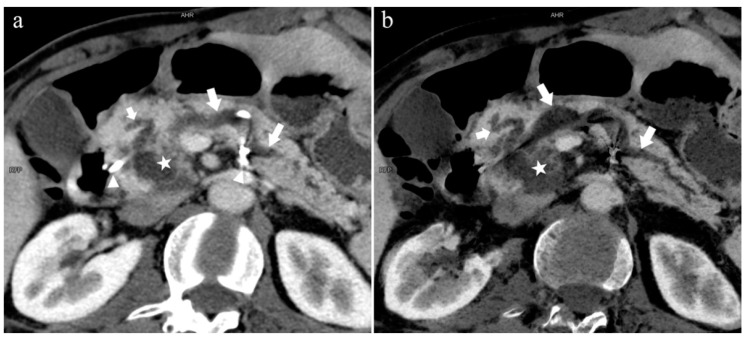
Type 3a. A 49-year-old male patient with IPMN. (**a**): MIP [45 keV MEI (+)] shows the enlarged head of the pancreas with tumor (pentagram), and tortuous dilation of MPD (long arrow) and APD (short arrow). The APD communicates with the front segment of the MPD without flowing into the MiP. The drainage tube (arrow) can be seen in the MPD. (**b**): MinIP [45 keV MEI (+)] reduces the interference of ductal artifacts and shows the course and more detail of the distal pancreatic duct. Postoperative pathology confirmed that the mass was IPMN.

**Figure 10:**
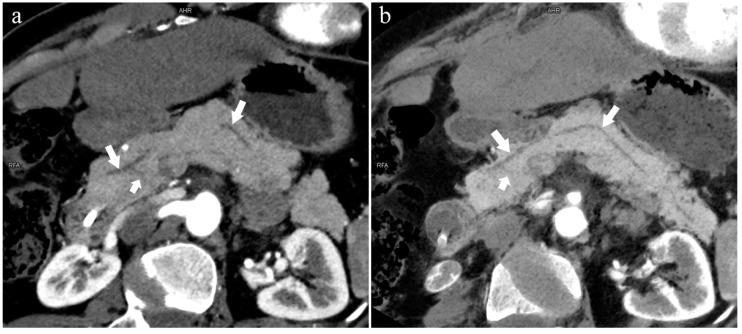
Type 2b. A 45-year-old female patient with Cholangitis. (**a**): MIP [45 keV MEI (+)] shows MPD (long arrow) flows into MiP and extends to the tail of the pancreas. The tiny APD (short arrow) communicates with the front segment of the MPD without flowing into the MP. (**b**): MinIP [45 keV MEI (+)] shows the overall configuration of the pancreatic duct and the thin duct in the tail. MPD refers to anatomically large and functionally dominant ducts.

There were 109 patients in the variant group. The PD (type 4) was the most common pancreatic variant (6.2%, 50/808), including type 4b (3.5%, 28/808, Figure 6), complete PD (type 4a) (1.6%, 13/808, Figure 7), and incomplete PD (type 4c) (1.1%, 9/808, Figure 8). The frequency of type 3a was 0.6% (5/808, Figure 9), and that of reverse PD (type 3c) was 3.6% (29/808). The frequency of type 2 was 2.4% (19/808), including type 2a (1.5%, 12/808) and type 2b (0.9%, 7/808, Figure 10). Anza pancreatica (type 5) was relatively rare (0.7%, 6/808), including type 5a (0.6%, 5/808) and type 5b (0.1%, 1/808).

### 4.3. Distribution of Pancreas-Related Diseases in the Normal and Variant Groups

Throughout the study, the number of patients in the normal group was significantly greater than that in the variant group, which was related to the low frequency of pancreatic duct variation.

There were 121 patients with AP in the two groups. In the variant group, a total of 27 patients had AP (22%, 27/121), of which the incidence of type 3c (33.3%, 9/27) and type 4a (18.5%, 5/27) are higher. There were 18 patients with CP, of which six patients (33%, 6/18) were in the variant group. Patients with autoimmune pancreatitis (AIP) only existed in the normal group (n = 4).

Overall, 99 patients (12.3%, 99/808) had pancreatic cancer and 43 patients (5.3%, 43/808) had other pancreatic tumors. Among the other pancreatic tumors, the incidence of intraductal papillary mucinous neoplasm (IPMN) (Figure 5 and Figure 9) was the highest at approximately 49% (21/43). In the “tumor at the opening of the pancreatic duct and common bile duct” group, there were 22 cases of DPC (2.7%, 22/808) and 46 cases of AC (5.7%, 46/808).

In addition, there were 239 patients (29.6%, 239/808) in the cholelithiasis and inflammation group and 141 patients (17.5%, 141/808) in the other group (no lesions in the biliopancreatic system and duodenum). Seventy-five patients (9.3%, 75/808) were in the biliary tumor group (except AC). These patients were included in the non-pancreas-related disease group.

As shown in Table 2, there were no statistical differences between the normal and variant groups in terms of PC, other pancreatic tumors, and AC. The distributions of the AP (*p* = 0.002), CP (*p* = 0.025), and DPC (*p* = 0.020) were significantly different in the normal and variant groups, respectively. In Table 3, except for patients with type 3c who had a correlation with AP (*p* = 0.014), there were significant differences between type 4a and AP, CP (*p* = 0.024, 0.025, respectively).

### 4.4. Comparison of the Sharpness of the Pancreatic Duct System between DECT and MRCP Images

We observed the low-keV DECT and MRCP images of 854 patients. Two patients with recurrence after surgical resection of pancreatic neoplasms were excluded, and 852 patients’ low-keV DECT and MRCP images were graded according to the sharpness of the pancreatic ducts for evaluation. A total of 44 patients had poor quality of either low-keV DECT or MRCP images, including 32 patients (3.8%, 32/852) with MRCP images Grade 1 and 12 patients (1.4%, 12/852) with low-keV DECT images Grade 1.

Furthermore, some patients had low-keV DECT images of Grade 2 but MRCP images of Grade 3, which could be diagnosed accurately by complementarity, and vice versa. Finally, there were 104 patients (12.2%, 104/852) with MRCP images of Grade 2 and 9 patients (1.1%, 9/852) with CT images of Grade 2.

The specific distribution is shown in Table 4.

In the 852 patients, there was a significant difference between the low-keV DECT and MRCP images in observing the pancreatic duct system (*p* < 0.05), and the mean rank of the former was higher than that of the latter (909.44 > 795.56). Thus, the sharpness of the pancreatic duct images of low-keV DECT is better than that of MRCP. Figure 6 shows that the reconstructed DECT images (MIP and MinIP, 45 keV) could show small ADP (Figure 6a,b), while this was not observed on MRCP (Figure 6c).

## 5. Discussion

In this study, we explored the pancreatic duct variation observed by low-keV MEI (+) of DECT and its relationship with related diseases. We found that pancreatic duct variation is associated with the occurrence and development of AP and CP. In particular, reverse PD (type 3c) and complete PD (type 4a) have a higher incidence of AP. It is worth noting that there was an association between DPC and pancreatic duct variation in our study. In addition, low-keV MEI (+) DECT is an effective method to observe the pancreatic duct system, and more patients can benefit from it.

### 5.1. Embryonic Development of the Pancreatic Duct

At approximately seven weeks of pregnancy, the ventral pancreatic bud rotates clockwise around the duodenum, merging with the dorsal pancreatic bud, which finally forms the pancreas [23]. The pancreas drains primarily through the ventral pancreatic duct, which enters the duodenum with the common bile duct at the major papilla. The dorsal pancreatic duct drains into the minor papilla [27].

Under normal circumstances, the main pancreatic duct (WirSong’s duct) runs along the full length of the pancreas and is connected to the accessory pancreatic duct (Santorini’s duct) at the head of the pancreas. However, with the growth and development of the body, APD may experience varying degrees of atrophy or even eventually disappear, which changes the morphology and drainage position of APD [8,28]. The most common types in the population are 1 and 3b, which is why they were set as the normal group.

### 5.2. Special Types of Pancreatic Duct Variation

#### 5.2.1. Pancreas Divisum (Type 4)

PD is the most common anatomical variation type of the pancreatic duct system and is caused by the failure of ventral and dorsal bud fusion [11,12]. In this study, the morbidity of type 4 was 6.2%, which is consistent with previous studies that showed rates of 1–8% in the ERCP examination and 4–14% in the autopsy population [29,30]. The most common type of PD was type 4b, with only MPD drainage to the MiP, followed by complete PD (type 4a); incomplete PD (type 4c) accounted for the least. This is not entirely consistent with the previous research of AnaDugic et al. [6]. One possible reason for this discrepancy is that some patients have inflammation or space-occupying in the head of the pancreas; thus, APD can be squeezed by surrounding edema parenchyma or tumor infiltration, which is an unavoidable influencing factor in the noninvasive examination.

#### 5.2.2. Ansa Pancreatica

Type 5, ansa pancreatica, was first proposed by Dawson and Longman in 1961. This is a very specific type of pancreatic duct variation that is rarely reported in the literature and is difficult to observe [31]. Some reports have put the incidence of this type at approximately 0.5–0.9% [32]. Type 5 accounted for 0.7% in our study, in which type 5a was more frequent than type 5b. Prasanna et al. [33] proposed that the distribution of the two is almost the same, which differs from our data. Ben Ismail, Imen et al. [32] pointed out that, due to the oblique connection between WirSong’s duct and Santorini’s duct, insufficient drainage of the MiP can easily occur, which increases the risk of AP recurrence. However, this hypothesis has not been confirmed. In the future, secretin-enhanced scans may be required to reveal fine-grained ADP variation, thereby increasing the accuracy and reliability of the study.

### 5.3. Correlation between Pancreatic Duct Variation and Related Diseases

#### 5.3.1. Duodenal Papillary Carcinoma (DPC)

The duodenal papilla is located on the medial wall of the descending duodenum and is the common opening of the common bile duct and pancreatic duct [34]. Because of the special anatomical location and physiological functions of the duodenal papilla, DPC is prone to duct obstruction in the early stage, and the “double duct sign” can be observed on imaging [35]. Of seven DPC patients in the variant group, there were five patients with Santorini’s duct as the dominant structure and opening in the MiP. Of the five patients, only the dorsal duct of the pancreas drained the entire pancreatic secretion to the MiP, resulting in stasis of pancreatic juice and eventual obstruction of the pancreatic duct. At the same time, MiP can be stimulated for a long time, which may lead to atypical cell proliferation and eventually constitute a tumor. In this study, it is surprising that there was a statistically significant association between the two groups in terms of the DPC, which further demonstrated our conjecture.

Theoretically, when the pancreatic duct is compressed or eroded by space-occupying lesions, the obstructed pancreatic juice may activate proteases causing inflammatory damage [36], especially in those variant types that are mainly drained by the MiP. However, with the low prevalence of this type of pancreatic duct variation in our study, it is necessary to further expand the sample size for further research in later studies.

Moreover, there were five cases of biliary tumors of types 4a and 4b. Although the pancreatic duct and the bile duct system originate from closely connected structures during embryonic development [37,38], there was no significant difference in the distribution of bile duct tumors between the two groups. This is consistent with Seungmin Bang et al. [39], who noted that there was no statistically significant correlation when examining the relationship between bile duct disease and pancreatic duct morphology.

#### 5.3.2. Pancreatitis

Kensuke Takuma et al. [27] suggested that complete PD may be the etiology of AP, CP, and dorsal pancreatic cancer. An important factor of complete PD promoting these diseases is dorsal pancreatic duct obstruction. We considered that, in type 4, only the dorsal pancreatic duct drains the pancreatic juice to the MiP. However, the MiP easily accumulates and stagnates due to the small physiological opening, which may lead to pancreatitis [40,41]. In this study, there were significant differences between AP (Figure 4), CP, and complete PD.

In the variant group, the incidence of AP in patients with type 3c was considerably higher than that in type 3a. Type 3c, also known as “reverse pancreas divisum”, does not communicate with the MPD but is open to the MiP, while type 3a is the opposite. In type 3c, there is a “vacuum” area between the MPD and ADP that may not be communicated, while type 3a cannot be drained directly by the MiP, but most areas can be drained indirectly by entering the MPD. This may be a reasonable explanation for this phenomenon. Table 4 also confirms our point of view. The frequency of type 2 was 2.4%, and AP accounted for 26% of type 2, which may have also been caused by the small orifice of the MiP drainage and the poor secretion flow [40,41].

Pathophysiologically, pancreatitis would cause dilation of the pancreatic duct, which can facilitate the drainage of pancreatic juice. The MiP is prone to edema and spasm once stimulated by inflammation, which leads to the opening being smaller or obstructed. In patients with pancreatitis and a pancreatic duct variation that drains mainly through the MiP (types 2, 4) the obstruction of pancreatic juice outflow may be aggravated, which deserves further observation and research.

Most CP patients experience acute inflammation, which results from a decrease in pancreatic exocrine and exocrine function [42]. Lew, Daniel, et al. [43] proposed that pancreatic duct anatomical variation, especially PD, constitutes one of the causes of recurrent AP and CP. In this study, there was a significant difference in the distribution of CP between the normal group and the variant group, and we believe that there is a correlation between CP and pancreatic duct variation. Pancreatic duct variation may therefore lead to abnormal drainage of pancreatic fluid and become an influencing factor of primary CP or lead to AP.

In patients with CP, in the presence of a prolonged inflammatory stimulus, the pancreatic parenchyma is prone to fibrosis and necrosis, and the pancreatic ducts appear to narrow, dilate, and distort [44]. It could be different from the changes in the pancreatic duct and the condition of pancreatic juice drainage in CP patients with pancreatic duct variation, which needs further research.

Meanwhile, four patients with AIP in this study all appeared in the normal group, and type 3b was more common. Because of the low incidence, it remains unclear whether there was a significant difference in its distribution. However, the occurrence of AIP is not caused by the anatomical variation of the pancreatic duct; rather, the continuous deposition of autoantibodies is the culprit [45,46]. Therefore, we believe that there is no correlation between pancreatic duct variation and autoimmune pancreatitis.

### 5.4. Comparison between Low-keV DECT and MRCP

Catheter and stent implantation through the wrong channel will damage the normal parenchyma of the pancreas, destroying the outflow channel of pancreatic juice, leading to the leakage of trypsin and a subsequent high risk of aggravating the patient’s condition [14,15,47]. Therefore, it is important to evaluate accurately the anatomy and opening of the pancreatic ducts before surgery. From another perspective, if the variation of the pancreatic duct can be found in time during the examination, and if patients can be informed about the matters needing attention and the corresponding risks before the occurrence of disease, related diseases can be prevented and clinicians can seek etiology and treatment to achieve a better long-term prognosis.

In the evaluation of the sharpness of pancreatic duct imaging, we found a significant difference between low-keV DECT and MRCP images (Table 3). The following reasons may be the key: First, the pancreatic duct system is not as developed as the bile duct system and the diameter of the pancreatic duct is smaller. Second, the examination requires patients to hold their breath so many times that some of them cannot cooperate accurately, resulting in a blurred image and even an inability to complete the examination. Perhaps most importantly, the observation of the pancreatic duct by MRCP requires the “cleanliness and tidiness” of the background plate; that is, the gastrointestinal tract is prepared, and there is no extensive inflammation or effusion in the abdominal cavity. Otherwise, the anatomical morphology of the pancreatic duct cannot be accurately observed. In clinical work, many patients are unable to meet the appropriate observation conditions.

In contrast, the emergence of low-keV DECT improves the contrast of the pancreatic duct and parenchyma with a lower radiation dose, and also reduces the dose of contrast media and the burden of renal excretion [21,22]. Moreover, it is not limited to any examination restrictions of MRCP, and the acceptance and completion of patients are greatly increased. Furthermore, CT is superior to MR in spatial resolution. The observation of the pancreatic duct and lesions can be more detailed with low-keV MEI (+) reconstructions. Inevitably, severe AP, pancreatic steatosis, and serious pancreatic atrophy affect the sharpness of the pancreatic duct on CT images.

There are some limitations in our study. First, if larger lesions or parenchymal edema and necrosis masked the anatomy of the pancreatic duct, the evaluation would be severely limited. MIP and MinIP images reconstructed at 40–50 keV could help to resolve this problem. Second, there was a certain deviation in the selection of patients because it was difficult to acquire normal and asymptomatic patients to complete both DECT and MRCP examinations in clinical work. Third, some types of pancreatic duct variation with low incidence required larger sample sizes to further analyze the relationship between those types and related diseases. Finally, our study mainly focused on the pancreatic duct, ignoring some variations in the bile duct system. However, we believe that the relationship between pancreatic duct variation and related diseases was accurately evaluated and that low-keV DECT had certain advantages in observing the pancreatic duct system; other studies will probably need to be performed to confirm this. Meanwhile, the reconstructed MEI (+) can be conveniently acquired by a post-processing workstation. Although that puts forward higher requirements for diagnosticians, it would not bring patients any additional medical expenses. In our opinion, using this technology when patients have pancreas-related diseases or are undergoing major biliopancreatic surgery is quite necessary.

## 6. Conclusions

In conclusion, our study suggested that pancreatic duct variation may be an influencing factor of AP, CP, and DPC. In the nonbiliary pancreatitis group, the distributions of PD (type 4) and reverse PD (type 3c) were the most significant. In addition, we found that low-keV DECT is an effective means to observe the pancreatic duct system.

## Figures and Tables

**Table 1:** Clinical Profile of the Participant.

	Normal	Variant	*p*
Age (years), mean ± SD	57.9 ± 14.5	59.3 ± 12.8	0.464 *
Gender, n (%)			
Male	368 (52.6)	58 (53.2)	0.913 ^#^
Female	331 (47.4)	51 (46.8)	
Pancreas-related diseases ^a^	289 (41.3)	64 (58.7)	0.001 ^#^
Non-pancreas-related diseases ^b^	410 (58.7)	45 (41.3)	

* Student *t* test, ^#^ Chi-square test. ^a^ Include groups of pancreatic neoplasms, nonbiliary pancreatitis, and tumors at the opening of pancreatic duct common bile duct. ^b^ Include groups of biliary system tumor, cholelithiasis with inflammation, and other. (The biliary tumor group does not include ampullary carcinoma.).

**Table 2:** Distribution of normal group and variant group in pancreas-related diseases.

	PN		NPB			Tumor at the Opening of Pancreatic Duct and Common Bile Duct	
	PC	Other Pancreatic Tumors	AP	CP	AIP	DPC	AC
Normal	89	38	94	12	4	15	37
Variant	10	5	27	6	/	7	9
Total	99	43	121	18	4	22	46
*p*	0.292 ^#^	0.713 ^#^	0.002 ^#^	0.025 *		0.020 *	0.214 ^#^

* Fisher’s exact test,^#^ Chi-square test. PN, pancreatic neoplasms; PC, pancreatic cancer; NBP, nonbiliary pancreatitis; AP, acute pancreatitis; CP, chronic pancreatitis; AIP, autoimmune pancreatitis; DPC, duodenal papillary carcinoma; AC, ampullary carcinoma. The biliary tumor group does not include AC.

**Table 3:** Correlation analysis between AP, CP, and type 3c, type 4.

	Normal Group	Type 3c	Type 4a	Type 4b	Type 4c
AP	94	9	5	4	3
Non-AP	605	20	8	24	6
*p*		0.014 *^#^	0.024 *^#^	0.782 *	0.113 *
CP	12	1	2	1	1
Non-CP	687	28	11	27	8
*p*		0.413 *	0.025 *^#^	0.402 *	0.154 *

* Fisher’s exact test; ^#^ *p* < 0.05, indicating statistical significance. AP, acute pancreatitis; CP, chronic pancreatitis.

**Table 4:** Graded 852 patients’ images quality of low-keV DECT and MRCP according to the sharpness of the pancreatic duct system.

	Grade of Subjective Image Quality			Mean Rank *	*p*
	G1	G2	G3		
Low-keV DECT	12	9	831	909.44	<0.05
MRCP	32	104	716	795.56	

* When *p* < 0.05, the larger the value of mean rank, the closer the variable is to a higher level. In this study, the higher the grade, the clearer the subjective image. DECT, Dual-energy computed tomography; MRCP, Magnetic resonance cholangiopancreatography.

## Data Availability

The datasets used and/or analyzed during the current study are available from the corresponding author on reasonable request. The data are not publicly available due to privacy or ethical concerns.

## Abbreviations

DECT = Dual-energy computed tomography; MRCP = Magnetic resonance cholangiopancreatography; ERCP = Endoscopic retrograde cholangiopancreatography; AP = acute pancreatitis; CP = chronic pancreatitis; DPC = duodenal papillary carcinoma; PD = Pancreas divisum; MPD = main pancreatic duct; APD = accessory pancreatic duct; MP = major papilla; MiP = minor duodenal papilla.
